# Intra-Bone Marrow Administration of Mesenchymal Stem/Stromal Cells Is a Promising Approach for Treating Osteoporosis

**DOI:** 10.1155/2019/4214281

**Published:** 2019-11-12

**Authors:** Hideki Agata, Yoshinori Sumita, Tatsuro Hidaka, Mayumi Iwatake, Hideaki Kagami, Izumi Asahina

**Affiliations:** ^1^Department of Regenerative Oral Surgery, Unit of Translational Medicine, Nagasaki University Graduate School of Biomedical Sciences, Nagasaki, Japan; ^2^Division of Molecular Therapy, Advanced Clinical Research Center, The Institute of Medical Science, The University of Tokyo, Tokyo, Japan; ^3^Basic and Translational Research Center for Hard Tissue Disease, Unit of Translational Medicine, Nagasaki University Graduate School of Biomedical Sciences, Nagasaki, Japan; ^4^Department of Oral Maxillofacial Surgery, Matsumoto Dental University, Nagano, Japan

## Abstract

Mesenchymal stem/stromal cells (MSCs) are known to be useful for treating local bone diseases. However, it is not known if MSCs are effective for treating systemic bone diseases, as the risk for mortality following intravenous MSC administration has hindered research progress. In this study, we compared the safety and efficacy of intra-bone marrow and intravenous administration of MSCs for the treatment of ovariectomy- (OVX-) induced osteoporosis. Cells capable of forming bone were isolated from the murine compact bones and expanded in culture. Relatively pure MSCs possessing increased potential for cell proliferation, osteogenic differentiation, and inhibition of osteoclastogenesis were obtained by magnetic-activated cell sorting with the anti-Sca-1 antibody. Sca-1-sorted MSCs were administered to OVX mice, which were sacrificed 1 month later. We observed that 22% of the mice died after intravenous administration, whereas none of the mice died after intra-bone marrow administration. With respect to efficacy, intravenous administration improved bone mineral density (BMD) by increasing bone mineral content without affecting bone thickness, whereas intra-bone marrow administration improved BMD by increasing both bone mineral content and bone thickness. These results indicate that intra-bone marrow administration of pure MSCs is a safer and more effective approach for treating osteoporosis.

## 1. Introduction

Mesenchymal stem/stromal cells (MSCs) have attracted much interest as potent somatic stem cells for use in regenerative medicine in various tissues/organs because of their ability to differentiate into multiple lineages (osteogenic, chondrogenic, adipogenic, myogenic, and neurogenic) [1, 2]. MSCs have recently gained attention as immunosuppressive cells that may be effective for treating immunological disorders such as graft-versus-host disease [3]. Therefore, MSCs are considered therapeutically useful cells, and their clinical use is expected to increase in the future. Because MSCs were originally identified as osteogenic stem/progenitor cells [4], potential therapeutic applications for bone tissue treatment have been extensively studied [5, 6]. Bone tissue engineering is the most successful application of MSCs, and transplantation of MSCs on scaffolds repairs bone defects more efficiently than artificial bone substitutes or even autologous bone grafting [5]. Accordingly, MSCs may also be applicable for treating systemic bone diseases such as osteoporosis.

In osteoporosis, the bone becomes porous and fragile because of an imbalance between bone formation and resorption [7, 8]. As osteoporosis is associated with aging and menopause, the number of osteoporosis patients is predicted to increase further as life expectancy increases. Therefore, the prevention and treatment of osteoporosis are of tremendous importance for achieving better health and longevity. Bisphosphonate (BP), which increases bone mineral density (BMD) by inducing apoptosis of osteoclasts, is currently used as the first-line therapy for the treatment of osteoporosis. However, long-term BP treatment causes severe suppression of bone turnover, which paradoxically increases fracture risks [7, 8]. In addition, BP-related osteonecrosis of the jaw (BRONJ) is a severe side effect of BP [7, 8]. For these reasons, there is a growing safety concern about BP treatment and the development of new osteoporosis treatments is needed.

Systemic administration of MSCs represents a new approach for treating osteoporosis [9], although only a limited number of studies have investigated its therapeutic effect in osteoporosis [9, 10]. Administration *via* the intravenous route, the most popular route of systemic administration, often leads to lethal pulmonary thromboembolism, which has hindered research progress [11]. Therefore, identification of a safe route for systemic administration of MSCs is important. A previous study reported that senile osteoporosis in SAMP6 (senescence-accelerated mouse prone 6) mice was successfully treated with bone marrow transplantation (intra-bone marrow injection of allogenic bone marrow cells) after irradiation [12]. Although treating osteoporosis with bone marrow transplantation is unrealistic, this study showed that MSCs, as well as hematolymphoid cells, could be efficiently transplanted by intra-bone marrow injection. Because intra-bone marrow injection has a low risk of pulmonary thromboembolism, we hypothesized that MSCs could be safely and more efficiently transplanted using this technique. Accordingly, we compared the safety and efficacy of intra-bone marrow and intravenous administration of MSCs for the treatment of ovariectomy- (OVX-) induced osteoporosis in mice.

## 2. Materials and Methods

### 2.1. Mice

Seven-week-old BALB/c mice (male and female) and 6-week-old BALB/CAJcl-nu/nu mice (female) were purchased from Clea Japan, Inc. (Tokyo, Japan). All experiments were approved by the Animal Ethics Screening Committee of the Institute of Medical Science, University of Tokyo.

### 2.2. Cryopreservation of Bone Marrow Cells and Cultivation of Compact Bone-Derived MSCs

After an intraperitoneal overdose administration of pentobarbital sodium, the femurs and tibiae of male mice were carefully dissected, and their epiphyses were removed. The bone marrow was then flushed out, collected in 50 mL centrifuge tubes (Becton Dickinson, Pharmingen, Franklin Lakes, NJ, USA), and centrifuged at 440 × *g* for 5 min at 4°C. After discarding the supernatants, bone marrow cells were suspended in 1 mL cell freezing medium (Bambanker™, NIPPON Genetics Co., Ltd., Tokyo, Japan) and cryopreserved at −80°C. In parallel, the bone cavities were washed thoroughly by drawing and expelling with a syringe. Thereafter, compact bones were cut into 0.5 to 1 mm^3^ sections, suspended in Dulbecco's phosphate-buffered saline (DPBS; Nissui Pharmaceutical Co., Ltd., Tokyo, Japan) containing 20% fetal bovine serum (FBS; JRS, Woodland, CA, USA) in the presence of 0.25% collagenase (Wako Pure Chemical Industries, Ltd., Osaka, Japan) in 50 mL centrifuge tubes, and incubated for 45 min at 37°C with shaking at 200 rpm. The tubes were then centrifuged at 440 × *g* for 5 min at 4°C, and the supernatants were discarded. The bone fragments (released cells and the bone sections) were suspended in serum-containing medium (*α*-MEM; Wako Pure Chemical Industries, Ltd.) supplemented with 10% FBS and antibiotics/antimycotics and recentrifuged under the same conditions. After discarding the supernatants, the bone fragments were resuspended in cell growth medium (serum-containing medium supplemented with 1 ng/mL basic fibroblast growth factor; Sigma-Aldrich, St. Louis, MO, USA), plated in 100 mm tissue culture dishes (TPP Techno Plastic Products AG, Trasadingen, Switzerland), and maintained in a 37°C, 5% CO_2_ incubator. After 1 week of culture without changing the medium, adherent cells (MSCs) were harvested with 0.05% trypsin-EDTA (Invitrogen, Carlsbad, CA, USA) and passaged at a density of 5 × 10^5^ cells per dish. MSCs were then cultured in the incubator until 80% confluent with a complete medium change twice a week.

### 2.3. Transplantation Experiment with Compact Bone-Derived MSCs

Because the ability to form bone *in vivo* is an important characteristic of MSCs, a transplantation experiment was performed. Compact bone-derived cells at passage 1 (2.5 × 10^5^ cells) were mixed with 25 mg *β*-tricalcium phosphate (*β*-TCP) granules (G1 type OSferion®; generously provided by Olympus Terumo Biomaterials, Tokyo, Japan) in a 14 mL polypropylene tube (Becton Dickinson) containing serum-containing medium. The next day, the culture medium was replaced with osteogenic induction medium (serum-containing medium with 10 nM dexamethasone (Sigma-Aldrich), 100 *μ*M ascorbic acid (Wako Pure Chemical Industries, Ltd.), 10 mM glycerol 2-phosphate disodium salt hydrate (*β*-glycerophosphate, Sigma-Aldrich), and 100 ng/mL recombinant human bone morphogenetic protein-2 (PeproTech, Rocky Hill, NJ, USA)). The culture medium was replaced with fresh medium on day 4. On day 7, after careful removal of the supernatant, 100 *μ*L of 10 mg/mL fibrinogen solution (bovine plasma fibrinogen (F8630, Sigma-Aldrich)) and 5 *μ*L of 100 U/mL thrombin solution (bovine plasma thrombin (T9549, Sigma-Aldrich)) were added to the cell-*β*-TCP mixtures to form a fibrin clot. Thereafter, each clotted cell mixture was transplanted into a subcutaneous space on the back of BALB/CAJcl-nu/nu mice anesthetized with pentobarbital sodium. Transplants were harvested 3 months after the operation and fixed in 10% buffered formalin, decalcified in Kalkitox® (Wako Pure Chemical Industries, Ltd.), and embedded in paraffin. Five-micrometer thick sections were cut and subsequently stained with hematoxylin and eosin.

### 2.4. Flow Cytometric Analysis

The expression of cell surface markers was analyzed with a FACS Aria flow cytometer (Becton Dickinson). Fluorescein isothiocyanate- (FITC-) conjugated, phycoerythrin-conjugated, allophycocyanin-conjugated, or peridinin-chlorophyll proteins with cyanine dye-conjugated antibodies targeted to CD11b, CD45, CD29, and Sca-1 (all from BioLegend, San Diego, CA, USA) were used for the analyses. First passage cells were detached with trypsin-EDTA, and 1 × 10^6^ cells were resuspended in 50 *μ*L ice-cold DPBS. Cells were then incubated with individual antibodies for 20 min on ice. Thereafter, cells were washed, resuspended in 200 *μ*L ice-cold DPBS, stained with propidium iodide, and analyzed. Data analysis was performed using FlowJo software (TreeStar, Inc., San Carlos, CA, USA).

### 2.5. Magnetic-Activated Cell Sorting (MACS) of Sca-1^+^ MSCs and CFU-f Assays

To isolate Sca-1^+^ MSCs, first passage cells were subjected to MACS using a MiniMACS Separator and MS columns (Miltenyi Biotec Inc., Bergisch Gladbach, Germany) according to the manufacturer's protocol. Cells were suspended in MACS buffer (DPBS containing 0.5% bovine serum albumin (Iwai Chemicals Company, Tokyo, Japan) and 2 mM EDTA (Invitrogen)) and filtered through a 70 *μ*m cell strainer (Becton Dickinson). After centrifugation at 300 × *g* for 10 min at 4°C, the supernatants were discarded, and the cells were resuspended in 40 *μ*L MACS buffer. The cells were then incubated with 10 *μ*L anti-Sca-1-biotin antibody (Miltenyi Biotec Inc.) for 10 min at 4°C. After incubation, the cells were washed by adding 2 mL MACS buffer and centrifuging at 300 × *g* for 10 min at 4°C. The cells were then resuspended in 100 *μ*L MACS buffer and incubated with 20 *μ*L anti-biotin MicroBeads (Miltenyi Biotec Inc.) for 15 min at 4°C. Thereafter, cells were washed by adding 2 mL MACS buffer and centrifuging at 300 × *g* for 10 min at 4°C. The cells were subsequently resuspended in 500 *μ*L MACS buffer and loaded onto columns that were surrounded by a magnetic field. After removal of unlabeled cells by repeated washing of the columns, Sca-1^+^ MSCs trapped in the columns were collected in 50 mL tubes and centrifuged at 440 × *g* for 5 min at 4°C. Finally, cells were suspended in cell growth medium, plated in 100 mm tissue culture dishes at a density of 5 × 10^5^ cells per dish, and cultured until 80% confluent with a complete medium change twice a week.

Simultaneously, the formation of colony-forming unit for fibroblasts (CFU-f) associated to the clonogenicity and stem cell activity was assessed on the nonsorted MSCs, Sca-1^−^ MSCs, and Sca-1^+^ MSCs, respectively. These 3 groups of MSCs were resuspended in serum-containing medium supplemented with 10 ng/mL basic fibroblast growth factor (Sigma-Aldrich), plated in 12-well plates at a density of 5 × 10^3^ cells per well or 1 × 10^4^ cells per well, and maintained in a 37°C, 5% CO_2_ incubator. After 14 weeks, cultures were washed 3 times with DPBS and then the colonies were stained with 0.5% crystal violet solution in methanol. Two examiners independently counted the colonies with 50 or more cells as CFU-fs in a blinded manner, and the percentage of stained area was analyzed by ImageJ software in 3 wells/specimen at each density of seeded cells, 3 specimens in each group.

### 2.6. Fluorescence Immunostaining of Sca-1

Sca-1-sorted MSCs were fixed with 4% paraformaldehyde, washed three times with DPBS, and treated with 100 mM glycine buffer. The cells were washed again three times and incubated with biotinylated antibodies against Sca-1 (1 : 200; BioLegend) at 4°C overnight. The next day, cells were rewashed three times and incubated with a streptavidin-FITC conjugate (1 : 800; BioLegend) for 20 min at room temperature. After three washes with DPBS, cells were counterstained with Vectashield mounting medium containing DAPI (Vector Laboratories Inc., Burlingame, CA, USA) and observed under a fluorescence microscope (Axio Observer Z-1 with an AxioCam HRm camera and AxioVision software (Carl Zeiss Japan, Tokyo, Japan)).

### 2.7. Analyses of Cell Proliferation and Osteogenic Differentiation Abilities

The potential for cell proliferation and osteogenic differentiation was analyzed as described elsewhere [13]. Nonsorted or Sca-1-sorted MSCs at passage 2 were plated into 24-well plates at a density of 2 × 10^4^ cells per well in serum-containing medium. The next day, cells were fed with noninduction medium (serum-containing medium) or osteogenic induction medium. The culture medium was replaced with fresh medium on day 4. On day 7, cell counting and quantitative alkaline phosphatase (ALP) assays were performed using a commercially available *p-*nitrophenyl phosphate tablet set (Sigma-Aldrich) and a cell counting kit-8 (WST-8®; Dojindo, Kumamoto, Japan). Briefly, 50 *μ*L WST-8 was added to each well containing 0.5 mL fresh medium and incubated for 60 min, and absorbance was read at 450 nm to assess cell numbers. After WST-8 analysis, each well was washed twice with DPBS, and 400 *μ*L *p-*nitrophenyl phosphate solution was added to each well. After incubation for 10 min at 37°C, the conversion to *p-*nitrophenol was stopped with 400 *μ*L 3 N NaOH, and the absorbance of *p-*nitrophenol was measured at 405 nm. ALP activity was expressed as *p-*nitrophenol absorbance (PNP (OD))/WST-8 absorbance (WST-8(OD)).

### 2.8. Analyses of Osteoclastogenesis Inhibition Potential

The potential to inhibit osteoclastogenesis was analyzed using a coculture assay. Cryopreserved bone marrow cells (BMCs) were thawed, suspended in serum-containing medium, and centrifuged at 440 × *g* for 5 min at 4°C. After discarding the supernatants, BMCs were resuspended in serum-containing medium, and the number of viable cells was counted using a Countess® Automated Cell Counter (Invitrogen) with fixed settings (sensitivity: 2; size gating: 10-60 *μ*m; circularity: 85%). BMCs were then seeded in the lower wells of 24-well plates (Becton Dickinson) at a density of 5 × 10^4^ cells per well, and a cell culture insert with 0.4 *μ*m diameter pores (Becton Dickinson) was placed in each well. Thereafter, nonsorted or Sca-1-sorted MSCs at passage 3 were seeded onto the cell culture inserts (the upper wells) of 24-well plates at a density of 2 × 10^4^ cells per well in serum-containing medium. Wells containing no cell culture inserts (no upper wells) were prepared as a control. The next day, 0.5 mL commercially available Mouse Osteoclast Culture Medium containing 50 ng/mL macrophage colony-stimulating factor (M-CSF) and 50 ng/mL receptor activator of nuclear factor kappa-B ligand (RANKL) (Primary Cell, Hokkaido, Japan) was added to the lower wells, and 0.5 mL serum-containing medium was added to the upper wells. The culture medium was replaced with fresh medium on day 4. On day 7, the supernatant in each well was collected and cryopreserved at −80°C for enzyme-linked immunosorbent assays (ELISA), and the plates were subjected to Tartrate-Resistant Acid Phosphatase (TRAP) staining.

### 2.9. TRAP Staining

TRAP staining was performed on day 7 of the coculture assays. After removing the cell culture inserts, the 24-well plates were washed once with DPBS and fixed with 4% paraformaldehyde for 5 min at room temperature. The plates were then washed three times with DPBS, and TRAP staining buffer from a commercially available kit (Primary Cell) was added to each well. After incubation for 30 min at 37°C, the plates were washed with distilled water to stop the reactions. The number of osteoclasts (TRAP-positive cells with ≥3 nuclei) in each well (cells/cm^2^) was assessed. The percentage of osteoclastogenesis inhibition (100 − (osteoclast number in experimental group/osteoclast number in control group) × 100) was subsequently calculated.

### 2.10. ELISA

The concentrations of osteoprotegerin (OPG), M-CSF, and RANKL in the supernatants of coculture assays were analyzed with commercially available ELISA kits (R&D Systems, Inc., Minneapolis, MN, USA) according to the manufacturer's protocol.

### 2.11. Systemic Administration of MSCs to OVX Mice

Syngeneic 7-week-old female mice underwent bilateral OVX or sham operation (Sham) under general anesthesia with pentobarbital sodium. OVX mice were divided into three groups as follows: OVX (control; no MSCs), OVX+Sca-1-iv (intravenous administration of Sca-1-sorted MSCs), and OVX+Sca-1-ib (intra-bone marrow administration of Sca-1-sorted MSCs). Intravenous administration was performed by injecting 5 × 10^5^ Sca-1-sorted MSCs in 100 *μ*L saline into a tail vein 2 months after OVX. Intra-bone marrow administration was performed by injecting 5 × 10^5^ cells in 20 *μ*L saline into the bone marrow cavity of the left tibia 2 months after OVX, as described elsewhere [11]. At 19 weeks of age (3 months after OVX), the mice were sacrificed and the right femurs were dissected, fixed in 70% ethanol, and subjected to micro computed tomography (Micro-CT) (Scan Xmate-L090; Comscantecno Co., Ltd., Kanagawa, Japan) and dual-energy X-ray absorptiometry (DEXA) (DCS-600EX-IIIR; Hitachi Aloka Medical, Ltd., Tokyo, Japan) analyses.

### 2.12. Statistical Analysis

Data are presented as the mean ± standard deviation. Two group comparisons were analyzed with the Student *t*-test. For multiple comparisons, data were analyzed with a one-way analysis of variance with post hoc comparison. Differences were considered statistically significant when *P* < 0.01 or *P* < 0.05.

## 3. Results

### 3.1. Characteristics of Compact Bone MSCs

**Figure 1:**
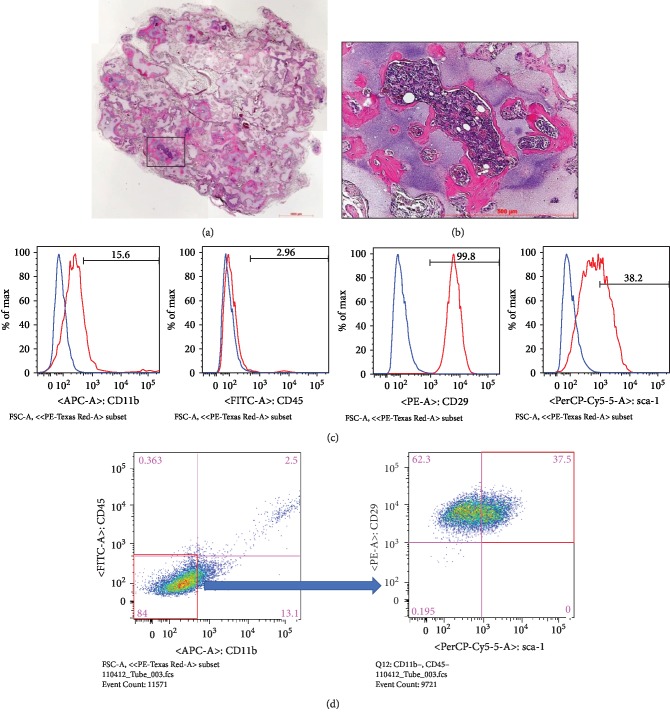
The characteristics of compact bone-derived MSCs. (a) Extensive new areas of bone formation were observed following MSC transplantation. (b) Higher magnification of the boxed area in (a). (c) Cell surface expression of CD11b, CD45, CD29, and Sca-1. (d) Expression of CD29 and Sca-1 in the nonhematopoietic cell population (CD11b^−^ and CD45^−^).

To investigate whether compact bone-derived cells are actually MSCs, the *in vivo* bone-forming ability, which is an important characteristic of MSCs, was analyzed with the transplantation experiment. Extensive new bone formation was observed in the transplants harvested after 3 months (Figure 1(a)). A higher magnification image is shown in Figure 1(b). Based on these results, compact bone-derived cells were used as MSCs in subsequent experiments. Although MSCs are comprised of a heterogeneous mixture of various cell populations including stem cells [11, 13], multipotent MSCs (i.e., relatively pure MSCs) in mice express CD29 and Sca-1 but not CD11b or CD45 [11, 13, 14]. Accordingly, we performed flow cytometric analyses of these cell surface markers in compact bone-derived MSCs. About 15% of the cells expressed CD11b, about 3% of the cells expressed CD45, almost 100% of the cells expressed CD29, and about 38% of the cells expressed Sca-1 (Figure 1(c)). Because CD11b and CD45 are markers of hematopoietic cells, we subsequently analyzed the expression of CD29 and Sca-1 in nonhematopoietic cell populations of compact bone-derived MSCs. As shown in Figure 1(d), almost 100% of nonhematopoietic cells (CD11b^−^, CD45^−^ cells) were positive for CD29, and about 38% were positive for Sca-1, indicating that almost all Sca-1^+^ cells were relatively pure MSCs (CD11b^−^, CD45^−^, CD29^+^, and Sca-1^+^) (Figure 1(d)).

### 3.2. Characteristics of Sca-1-Sorted MSCs

**Figure 2:**
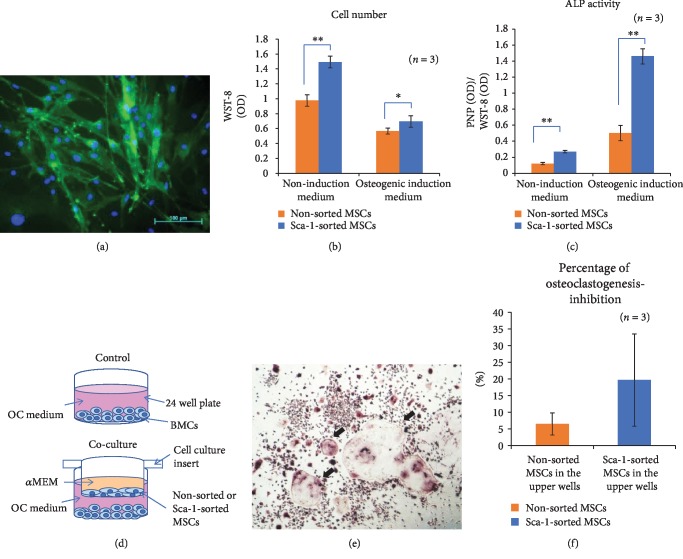
The characteristics of Sca-1-sorted MSCs. (a) Fluorescence immunostaining of Sca-1. (b) Cell number analysis after 1 week of culture. (c) ALP assay after 1 week of culture. (d) A schematic of the coculture assay to assess the ability of nonsorted or Sca-1-sorted MSCs to inhibit osteoclastogenesis. BMC: bone marrow cells; OC: osteoclasts. (e) After 1 week of coculture, the lower wells were subjected to TRAP staining. Arrows indicate osteoclasts. (f) The percentage of osteoclastogenesis inhibition was greater with Sca-1-sorted MSCs, although the difference was not statistically significant. ^∗^*P* < 0.05. ^∗∗^*P* < 0.01.

Accordingly, magnetic-activated cell sorting (MACS) of Sca-1^+^ cells was performed to obtain relatively pure MSCs. Fluorescent immunostaining of Sca-1-sorted MSCs showed that almost all of the cells were positive for Sca-1 (Figure 2(a)). Moreover, regarding the clonogenicity and stem cell activity of Sca-1-sorted (Sca-1^+^) MSCs, these cell populations increased the number of CFU-f and its positive area significantly compared with nonsorted MSCs or Sca-1^−^ MSCs (Supplemental [Supplementary-material supplementary-material-1]–[Supplementary-material supplementary-material-1]). Then, cell proliferation and ALP activity were quantitatively analyzed after 1 week of culture in noninduction medium or osteogenic induction medium. Sca-1-sorted MSCs showed higher cell numbers in both noninduction and osteogenic induction medium than nonsorted MSCs (Figure 2(b)). ALP activity was also greater in Sca-1-sorted MSCs in both noninduction and osteogenic induction medium (Figure 2(c)). These results indicate that Sca-1-sorted MSCs were relatively pure with superior growth and osteogenic differentiation abilities. Subsequently, the ability of nonsorted or Sca-1-sorted MSCs to inhibit osteoclastogenesis was analyzed using a coculture assay, as described in the schematic (Figure 2(d)).

**Figure 3:**
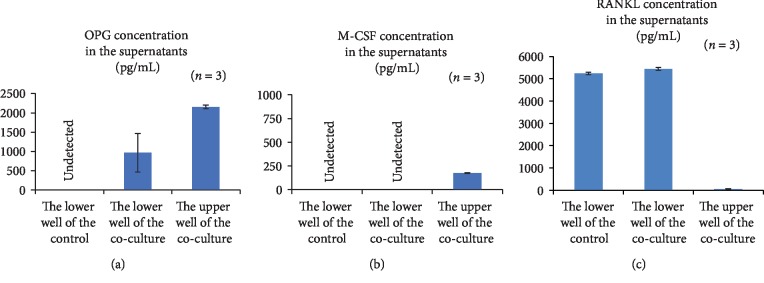
The supernatant collected from each well of the coculture experiment was subjected to ELISA for OPG (a), M-CSF (b), and RANKL (c).

After 1 week of culture, the supernatant from each well was collected for ELISA, and the plates were subjected to TRAP staining. TRAP-positive cells with ≥3 nuclei were counted as osteoclasts (Figure 2(e)), and the percentage of inhibition of osteoclastogenesis was calculated. As shown in Figure 2(f), the percentage of osteoclastogenesis inhibition was higher in Sca-1-sorted MSCs, although the difference was not statistically significant. ELISA for osteoprotegerin (OPG), macrophage colony-stimulating factor (M-CSF), and receptor activator of nuclear factor kappa-B ligand (RANKL) showed that Sca-1-sorted MSCs actively released the osteoclastogenesis inhibitory factor OPG; however, little or no release of the osteoclastogenesis promoting factors M-CSF and RANKL was observed (Figures 3(a)–3(c)). These results indicate that compared to nonsorted MSCs, Sca-1-sorted MSCs possessed a greater ability to inhibit osteoclastogenesis in addition to their superior growth and differentiation abilities.

### 3.3. Effects of Systemic Administration of Sca-1-Sorted MSCs in OVX Mice

**Figure 4:**
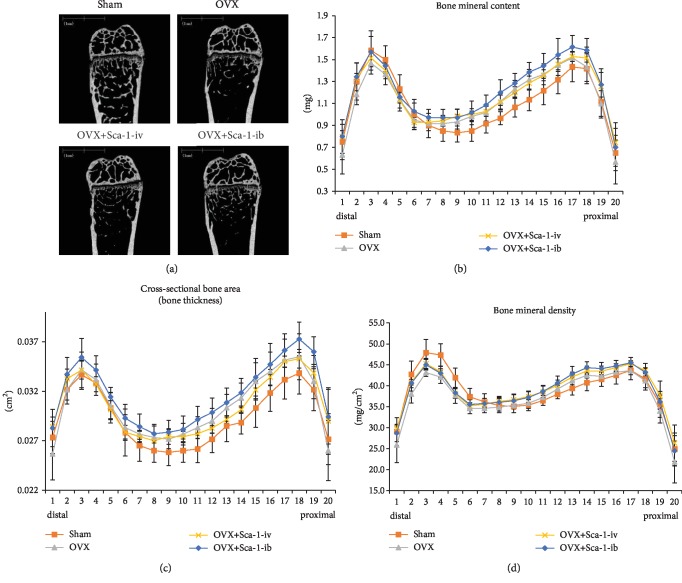
The effect of systemic administration of Sca-1-sorted MSCs *via* an intravenous or intra-bone marrow route (OVX+Sca-1-iv or OVX+Sca-1-ib mice, respectively) on bone parameters. (a) Representative micro-CT photographs of the right femurs of Sham, OVX, OVX+Sca-1-iv, and OVX+Sca-1-ib mice. (b) DEXA analysis of bone mineral content. (c) DEXA analysis of cross-sectional bone area (bone thickness). (d) DEXA analysis of BMD.

To investigate the potential application for treating osteoporosis, Sca-1-sorted MSCs were systemically administered to OVX mice 2 months after OVX *via* an intravenous or intra-bone marrow route (OVX+Sca-1-iv or OVX+Sca-1-ib mice, respectively). One month after cell administration, the mice were sacrificed for analysis. Sham-operated (Sham) mice and OVX mice without treatment were also analyzed. Two of the OVX+Sca-1-iv mice died shortly after cell administration due to pulmonary embolism, resulting in a mortality rate of 22%. Conversely, all OVX+Sca-1-ib mice survived until the end of the experiment, indicating that the intra-bone marrow route is a safer approach for administering MSCs. Because both Sham and OVX mice showed a 100% survival rate, the final number of mice in each group was as follows: Sham (6/6), OVX (6/6), OVX+Sca-1-iv (7/9), and OVX+Sca-1-ib (7/7). Micro-CT analyses of right femurs showed that OVX mice developed osteoporosis due to accelerated trabecular bone resorption (Figure 4(a)). Although both OVX+Sca-1-iv and OVX+Sca-1-ib mice also showed lower amounts of trabecular bone than Sham mice, their bone conditions appeared better than those of OVX mice (Figure 4(a)). Accordingly, we subsequently performed dual-energy X-ray absorptiometry (DEXA) analyses (slice thickness: 1 mm; speed: 12.5 mm/s; from distal to proximal) to quantitate the changes in bone mineral content, cross-sectional bone area (bone thickness), and bone mineral density (BMD). Compared with OVX mice, OVX+Sca-1-iv mice showed slightly higher bone mineral content from slice-points 1 to 5 and comparable mineral content from slice-point 6 onward, whereas OVX+Sca-1-ib mice showed higher mineral content in all slice-points (Figure 4(b)). Thus, both intravenous and intra-bone marrow administration of Sca-1-sorted MSCs increased femoral bone mineral content, although intra-bone marrow administration was more effective. Analyses of bone thickness also indicated that intra-bone marrow administration was more effective. OVX+Sca-1-iv mice showed comparable bone thickness from slice-points 1 to 10 and lower bone thickness from slice-point 11 onward, whereas OVX+Sca-1-ib mice showed greater bone thickness in all slice-points compared with OVX mice (Figure 4(c)). As for BMD, both OVX+Sca-1-iv and OVX+Sca-1-ib mice showed greater values than OVX mice at all slice-points, although their BMDs were still lower than those of Sham mice from slice-points 1 to 7 (Figure 4(d)).

## 4. Discussion

This study examined the safety and efficacy of intra-bone marrow administration of pure MSCs for the treatment of OVX-induced osteoporosis in mice. Our outcomes were (1) Sca-1-sorted MSCs possessed greater potential for cell proliferation, osteogenic differentiation, and osteoclastogenesis inhibition, (2) intra-bone marrow administration was safer than intravenous administration for MSC transplantation, and (3) intra-bone marrow administration of Sca-1-sorted MSCs improved BMD by increasing both bone mineral content and bone thickness. These outcomes suggest that delivery of pure MSCs via the intra-bone marrow route is a promising treatment strategy for osteoporosis.

Regarding the first outcome, an ideal osteoporosis therapy should have both anabolic and antiresorptive effects because osteoporosis is caused by an imbalance between bone formation and resorption. Therefore, we investigated whether pure MSCs exhibit superior characteristics in both anabolic and antiresorptive effects *in vitro*. BP therapy, the current first-line therapy for osteoporosis, produces only the antiresorptive effect. Thus, long-term use of BP leads to severe suppression of bone turnover, which paradoxically increases fracture risks [7, 8] and may also lead to serious side effects, such as BRONJ or atypical femoral fractures [15–17]. In contrast, parathyroid hormone (PTH) therapy only produces the anabolic effect. As PTH does not affect osteoclast activity, abrupt termination of PTH leads to a rapid decline in BMD. Unfortunately, the duration of this therapy is limited to 2 years over a patient's lifetime and patients must continue using other osteoporosis therapies after PTH therapy is no longer available [15]. Therefore, treatments lacking either anabolic or antiresorptive effects are associated with severe side effects. Viewed in this light, MSCs represent a useful tool for osteoporosis treatment because both anabolic and antiresorptive effects can be expected following transplantation of MSCs. In the bone environment, it is well known that bone marrow MSCs not only serve as a source of osteoblasts but also support osteoclast differentiation [14]. More recently, systemic transplantation of MSCs has been reported to recover the impaired function of recipient bone marrow MSCs and to regulate osteogenesis and osteoclastogenesis via IL-17 suppression in a secondary osteoporosis mouse model [18]. Accordingly, Sca-1-sorted MSCs are expected to replicate this remedy because they possess the ability to inhibit osteoclastogenesis by releasing OPG in addition to their abundant ability for osteogenic differentiation, compared with nonsorted MSCs.

With regard to *in vivo* outcomes, the mechanism of BMD improvement was interestingly very different between OVX+Sca-1-ib and OVX+Sca-1-iv mice, although BMD was comparable at all slice-points. As mentioned above, the improvement in BMD in OVX+Sca-1-iv mice was due to an increase in bone mineral content without any obvious change in bone thickness, whereas the improvement in OVX+Sca-1-ib mice was due to an increase in both bone mineral content and bone thickness. Therefore, Sca-1-sorted MSCs improved both the internal and external bone environment when administered *via* an intra-bone marrow route. In previous research, it has been demonstrated that MSCs from exfoliated deciduous teeth induce activated T-cell apoptosis in OVX mice *via* a FasL/Fas pathway when administered *via* an intravenous route [19]. Furthermore, this phenomenon resulted in immune tolerance and led to reduced osteoclast activation in OVX mice. On the other hand, it has been shown that bone marrow administration *via* an intra-bone marrow route could normalize the imbalance between osteoclastogenesis and osteoblastogenesis by improving the hematopoietic system as well as the bone marrow microenvironment in senile osteoporosis mice (SAMP6 mice) [12]. These facts indicate that the intra-bone marrow route is more preferable to restore bone marrow function *via* the anabolic and antiresorptive effects of administered MSCs, particularly Sca-1-sorted MSCs. These cells are expected to display both osteogenic differentiation and inhibition of osteoclastogenesis in *in vivo* bone environments. In addition, a large number of intrabone-administered MSCs should remain in the bone environment, whereas more than 90% of MSCs become trapped in the lungs and other tissues when they are infused by intravenous injection [20]. Furthermore, tibial administration, *via* the intra-bone marrow route, has been shown to rapidly accelerate the proliferation of donor-derived progenitor cells, even in the bone marrow of noninjected femur [21]. Therefore, intra-bone marrow administration of Sca-1-sorted MSCs may be a more focused remedy because Sca-1-sorted MSCs possess a greater potential for cell proliferation. Indeed, regarding the clinical use of MSCs, it has been concerned the function of MSCs declines with age or culture expansion [22–24]. Such senescent MSCs are known to show not only the decreased proliferation and differentiation potentials but also the reduced migratory, homing, and immune modulation abilities as an explant. From this aspect, Sca-1 sorting may be beneficial to avoid the senescent cells and preserve the therapeutic potentials of MSCs because Sca-1-sorted MSCs displayed the higher proliferative and clonogenic potentials than those of nonsorted MSCs.

## 5. Conclusions

Systemic administration of pure MSCs without senescent cells is a promising new approach for treating osteoporosis; however, effective and practical conditions for systemic administration remain unknown. This study presented that intra-bone marrow administration may be superior to intravenous administration in terms of safety and efficacy for cell therapy with Sca-1-sorted MSCs. However, further investigations are required to elucidate the optimal conditions, because the BMD of mice that received intra-bone marrow administration (cell number: 5 × 10^5^ cells; frequency: single administration) was still lower than that of Sham mice. Administration of adequate cell numbers at an appropriate frequency will enable further improvements in BMD.

## Data Availability

The data used to support the finding of this study are included within the article.

## Supplementary Materials

Supplementary Materials Supplemental Figure 1: the characteristics of Sca-1-sorted MSCs. Colony-forming unit for fibroblast (CFU-f) assay in culture of nonsorted MSCs, Sca-1-negative MSCs, and Sca-1-positive MSCs. (a) Crystal violet staining for CFU-fs. The density of 5 × 103 cells and 1 × 104 cells per well was assessed for each MSC fraction. (b) The total numbers of CFU-fs. (c) Total CFU-f-positive areas relative to culture dish area (^∗^*P* < 0.01).
