# Supplementary material for: Intra-Bone Marrow Administration of Mesenchymal Stem/Stromal Cells Is a Promising Approach for Treating Osteoporosis
Source: Stem Cells Int. 2019 Nov 12;2019:4214281. doi: 10.1155/2019/4214281 (PMC6875206; doi:10.1155/2019/4214281)
Supplement: Supplementary Materials — Supplemental Figure 1: the characteristics of Sca-1-sorted MSCs. Colony-forming unit for fibroblast (CFU-f) assay in culture of nonsorted MSCs, Sca-1-negative MSCs, and Sca-1-positive MSCs. (a) Crystal violet staining for CFU-fs. The density of 5 × 10^3^ cells and 1 × 10^4^ cells per well was assessed for each MSC fraction. (b) The total numbers of CFU-fs. (c) Total CFU-f-positive areas relative to culture dish area (∗P < 0.01). [file 4214281.f1.docx]

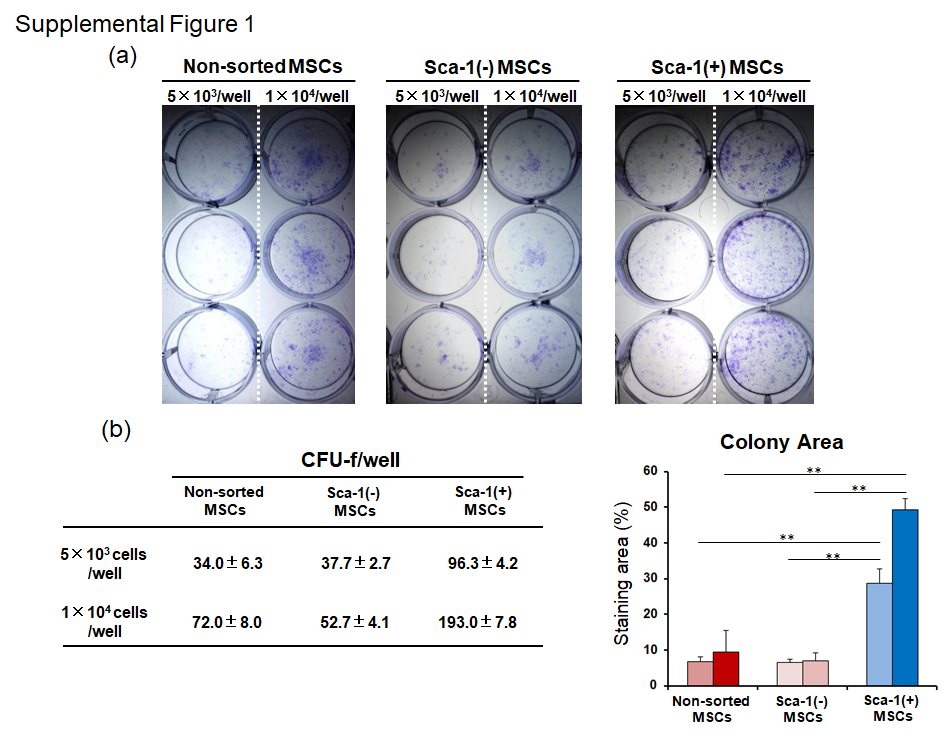


*

*

(n=3)

(n=3)

**SUPPLEMNRAL FIGURE 1:** The characteristics of Sca-1-sorted MSCs. Colony-forming unit for fibroblasts (CFU-f) assay in culture of non-sorted MSCs, Sca-1 negative MSCs, and Sca-1 positive MSCs. (a) Crystal violet staining for CFU-fs. The density of 5 × 10^3^ cells and 1 × 10^4^ cells per well was assessed for each MSC fraction. (b) The total numbers of CFU-fs. (c) Total CFU-f positive areas relative to culture dish area. *P < 0.01
